# Perceptions of Mental Health Professionals on Nutritional Psychiatry as an Adjunct Treatment in Mainstream Psychiatric Settings in New South Wales, Australia

**DOI:** 10.7759/cureus.56906

**Published:** 2024-03-25

**Authors:** Junaid Minhas, Jamee C Mcbride

**Affiliations:** 1 Psychiatry and Behavioral Sciences, Oceania University of Medicine, Sydney, AUS; 2 Emergency Department, Dubbo Base Hospital, Dubbo, AUS

**Keywords:** psychiatry and mental health, nutrition education, nutritional interventions, nutrition and mental health, nutritional psychiatry

## Abstract

Background

Nutritional psychiatry refers to the practice of using food, or nutrition, as alternative or complementary treatment for mental health disorders. It is a growing area of research that has shown links between the biological processes in the gut and how the food we consume can impact cognitive function, which then can impact our mood and behaviour. However, there is a lack of understanding on the knowledge and education of nutritional psychiatry in mental health clinicians, and further, how nutritional psychiatry, if at all, is practised in psychiatric clinical settings. Therefore, this study aims to investigate the perceptions, knowledge, and education of mental health professionals within the state of New South Wales, Australia regarding their clinical practice and knowledge of nutritional psychiatry.

Methods

In this cross-sectional study, a self-administered structured questionnaire created by the authors was successfully completed by 40 mental health professionals (mental health nurses, occupational therapists, psychologists, medical officers, and other allied health workers) who were working in New South Wales, Australia. 49 questionnaires were attempted with nine excluded due to these being predominantly incomplete, including demographic data only or empty, ensuring integrity of the remaining data and analysis. The questionnaire aimed to uncover their perceptions, knowledge, and education in nutritional psychiatry and its role in mental health settings. The questionnaire included 16 questions that covered various themes such as the frequency and importance of discussing nutrition with patients, nutrition referrals for issues regarding nutrition, the value of including nutritional psychiatry in their clinical practice, and clinician training and willingness to train or gain further education in nutritional psychiatry. Data was analysed through a series of frequency tables to categorise patterns and identify patterns through the use of descriptive statistics in our analysis of the distribution of attitudes and practices among mental health professionals regarding nutritional psychiatry.

Results

A total of 40 mental health clinicians successfully completed the survey and their results were analysed. The results suggest that most clinicians (85% to 93%) recognised the importance of nutrition in mental health, however, revealed various barriers which hindered their clinical practice such as a lack of time, knowledge, and access to nutritional services. Further, 54% of clinicians rated their knowledge of nutritional psychiatry as low, however, 92% revealed that they would attend nutritional psychiatry training if given the opportunity. Additionally, the clinicians reported varied levels of education in nutrition, with 43% reporting no education at all on nutrition in their preclinical studies.

Conclusions

Clinicians recognise the importance of nutrition in mental health settings, but revealed lack of time, knowledge, and nutritional services as barriers in clinical practice. Further, clinicians reported a lack of pre-clinical education on nutrition together with an overwhelming interest in engaging with nutritional psychiatry education if it were made available. These conclusions provide important insights for higher education and health policy.

## Introduction

The emerging field of nutritional psychiatry has expanded in the academic discourse throughout the last decade, where an increasing number of nutrient-focused intervention studies are completed and which demonstrates potential improvement in mental health outcomes for patients [1]. It refers to the practice of using food (or 'nutrition') as an alternative or complementary treatment for mental health disorders [2,3]. It is quite an important development as mental illness is one of the leading causes of disability globally, with over $8 trillion estimated in lost output due to these disorders [4]. Current treatment regimens often involve pharmacotherapy and psychotherapy, however, the recent discourse suggests that the increase in use of psychotropics isn’t necessarily reducing the burden of disease amongst affected populations. Indeed, the incidence of mental illness is increasing despite the increased use of these therapies [5]. Interestingly, the majority of the public believe that mood disorders such as depression are caused by reduced serotonin levels or activity (which then are justified in the use of antidepressants), however, this has been recently debunked by Moncrieff and colleagues, who declared that "there is no convincing evidence that depression is associated with, or caused by, lower serotonin concentrations or activity", thus allowing further research in other potential pathological factors such as diet [6].

Diet has been shown to be a modifiable risk factor for mental illness, such as anxiety and depression, particularly as diet has been linked to the biological pathways of inflammation, oxidative stress, gut microbiome and neuroplasticity [4]. This is particularly relevant in nutritional psychiatry as lower levels of antioxidants and vitamins E and C have been found in populations where depression is medically diagnosed against controlled populations [7,8]. Similarly, neurogenesis has been associated with mood regulation and there where brain chemistry is altered, mental illness can be clinically associated and some evidence has demonstrated that brain-derived neurotrophic factor (BDNF) can be improved through diet, therefore limiting or improving mental illness [6]. Furthermore, increased fat intake can cause changes within the gut microbiome, consequently changing the way gut flora signalling is understood by the brain [5,7,9]. This works via a complex system where metabolites, in breaking down food in the gut, make their way into the intestinal wall and interact with the enteric nervous system, which consequently transmits information to the brain and may also interact with immune cells, therefore potentially triggering immune responses [9]. This demonstrates a complex interplay between diet and inflammation through the gut microbiome, which consequently can lead to the pathogenesis of depression and anxiety, with some studies demonstrating links between high-fat food intake and dysregulation within this gut microbiome and, therefore, impacting this important brain signalling mechanism [9]. Some studies have conversely shown that individuals who consume the Mediterranean diet (a diet high in fish, fruits, and healthy vegetable oils) are at a reduced risk of developing mental illness such as depression [10].

In addition, recent literature has suggested that improving one’s diet quality could potentially be used as a treatment for mood disorders such as major depression [2,7,11]. One such trial, the SMILES trial published in 2017, assessed the merits of diet intervention in participants who were clinically diagnosed with major depression [12]. The study found that one-third of the participants, after 12 weeks of diet intervention, were in remission compared with 8% who were provided with social support only. Similarly, another recent study demonstrated a correlation between nutritional intake or supplementation of unsaturated fatty acids (omega-3), fish and zinc in improving mood disorder symptoms, particularly bipolar disorder [13]. Other studies have identified that supplementation or increased intake of omega-3, B vitamins, vitamin D, and other nutrients may provide neurochemical modulation which benefits the treatment and management of mood disorders [11]. These findings offer a potential new treatment for depressive mood disorders and therefore represents an interesting insight in the future role of nutritional psychiatry in clinical practice. However, there has been a lack of discourse on the perceptions and educational knowledge of clinicians regarding nutritional psychiatry within mental health settings. 

The rationale for our study is twofold: first, it addresses the growing discourse of nutritional psychiatry and, in particular, the link between diet and mental health outcomes within clinical psychiatric settings; second, our study investigates the education and training of mental health professionals in nutrition, their readiness and willingness in providing nutritional interventions as part of their clinical practice. Therefore, this study aims to investigate the perceptions, knowledge, and education of mental health professionals within the state of New South Wales, Australia, on nutritional psychiatry. 

## Materials and methods

Study design

This cross-sectional study utilised an opt-in methodology to collect quantitative and qualitative data from mental health professionals within New South Wales, Australia. Questionnaire data was obtained from three psychiatric facilities that provided both in-patient and out-patient services within New South Wales. The target population for the research were a range of mental health professionals, with a focus on obtaining data from mental health nurses, occupational therapists, psychologists, medical officers and other allied mental health professionals who work in the Australian mental health community. Therefore, a purposive sampling technique was utilised to recruit participants to the study which was an opt-in study. We made contact with various mental health centres and clinics to assist in distributing the questionnaire via a printable format or QR code to any mental health professionals who were interested in partaking in the study through April and May 2023, with three psychiatric facilities assisting in distribution of the questionnaire. The ethical approval for this survey was granted by the Institutional Review Board of the Oceania University of Medicine, Sydney, Australia (approval number 22-0322JM), with participant completion of the survey (as it was self-administered) considered as implied consent. As this was an opt-in survey, the size of the sample was determined by participant intake, with 49 participants initially accessing the questionnaire and 40 participants completing the questionnaire and therefore included in the study. Therefore, the response rate was 81.63% based on the questionnaires attempted/accessed and those that were completed. 

Inclusion criteria 

To minimise bias and increase reliability of results, an inclusion criteria was developed for this study. As this is an Australia-focused study, inclusion required participants to be primarily located within New South Wales, Australia, and more specifically, health professionals who specialise and/or work within the mental health clinical setting. Furthermore, inclusion into the study required respondents to be practising or retired mental health professionals (mental health nurses, occupational therapists in a mental health setting, psychologists, medical officers and other allied mental health professionals) within the mental health community in New South Wales, Australia.

Exclusion criteria 

As this study is focused on mental health professionals and associated allied health mental health clinicians, clinicians from other occupational fields or specialities were excluded from this study. Clinicians were given access to the questionnaire through the three clinical sites that were psychiatric. Thus, we ensured that participants in our study were clinicians working in a mental health setting. Furthermore, to obtain reliable data, clinicians who have not worked within the Australian, and more specifically New South Wales, health districts were excluded as target participants for this study. Nine respondents were excluded as their questionnaire responses were incomplete or invalid due to the majority (90% or more) of the questions remaining incomplete, or not attempted. 

Instrument 

A self-administered questionnaire consisting of 16 questions was provided to mental health practitioners within New South Wales and addressed various aspects of nutrition and mental health. These questions were segregated to gather data on different objectives, including professional background and setting, experience and attitudes, educational knowledge, clinical practice and implication, barriers, and value of education. These included questions that explored the frequency and importance of communicating with patients about their diet, investigating the referral process where nutritional deficiencies were evident, the value of incorporating nutritional psychiatry within the patient consultation and if the respondents had completed any training and/or show a willingness to complete training in nutritional psychiatry. The structured questionnaire utilised a variety of question types to gather quantitative (frequency) and qualitative responses, including a range of multiple choice questions which asked demographic information and allowed respondents to measure their level of agreement or disagreement to certain statements. In addition, a numerical rating scale from 1 (never/low) to 10 (always/high) was also utilised to measure respondent response to certain prompts or statements, therefore allowing a quantitative analysis of the data. We interpreted these scales as 1 to 3 being low frequency, 4 to 6 as moderate frequency, and 7 to 10 as high frequency. Furthermore, at the end of the questionnaire, respondents were allowed the opportunity to respond to certain prompts (their reflections and experiences), all of which were optional and not mandatory to increase the likelihood of collection of qualitative data. 

Further, to ensure content validity of our questionnaire, the questions were designed to encompass a variety of topics relevant to nutritional psychiatry and mental health. Content within the questionnaire was based on a performed literature review to ensure that aspects queried were relevant to the field and discourse. Further, the questionnaire was validated through a review and pilot by a small group of mental health professionals to ensure that the questions were interpreted as intended and that the questionnaire was measuring the data of interest, with revisions made based on this initial feedback. Furthermore, this survey study followed a similar research design by Michielson & De Sme who developed an instrument to assess clinician attitudes towards nutritional psychiatry that also had a similar survey response rate [14]. 

Ethical considerations 

The questionnaire developed for this research paper was designed with anonymity in mind. All participants were informed prior to the start of the questionnaire that no identifiable data will be collected and further, all results will be confidential with no identifiable information presented within the research report. Participation was entirely voluntary and as the questionnaire was self-administered, completion of the questionnaire was considered as implied consent from the respondent. 

Data analysis 

Questionnaire results were transferred to a Microsoft Excel document, which then allowed for the creation of descriptive statistics (frequency) to summarise the responses. The frequency tables which were created provided the number and percentage of respondents for each question, allowing for ease of understanding and analysis of the respondent data.

## Results

The initial distribution of our opt-in questionnaire yielded 49 responses. However, nine returned questionnaires that were accessed but deemed incomplete, with the majority (90% or more of the questions not attempted) and, therefore, excluded from the final analysis, which led to a final sample size of 40 respondents. The response rate was 81.63% based on the questionnaires attempted/accessed and those that were completed. This reduction was necessary to ensure the completeness of the questionnaire dataset, and although this resulted in a smaller sample size, the data that was retained for analysis was of high quality and remained relevant for our research questions. After the data was collected, the responses from the questionnaire were transferred from Google Forms to a Microsoft Excel document. Descriptive statistics were then generated using the built-in functions of Excel, including measures of frequency, percentages, and other functions to assess the distribution of the data. Respondents' demographic data, including profession, employment type, time employed were summarised using frequencies and percentages.

Demographic statistics

Table 1 below displays descriptive statistics regarding the demographic statistics as outlined by the respondents within the questionnaire. 

**Table 1:** Frequency in distribution and percentages of respondents’ demographic data (Q1-3) Total number of participants (N) = 40

Clinical Role	Frequency (n)	Percent (%)
Registered/enrolled Nurse	25	62.5%
Other allied health professional not listed	6	15%
Psychologist	4	10%
Psychiatry Registrar/psychiatrist	5	12.5%
Institution Recently/Last Worked at		
Hospital/inpatient department	31	77.50%
Mental health outpatient department	3	7.50%
Day clinic	3	7.50%
Hospital/inpatient department, mental health outpatient department	3	7.50%
Clinical Experience		
21+ years	7	17.50%
16 to 20 years	1	2.50%
11 to 15 years	4	10%
6 to 10 years	8	20%
2 to 5 years	17	42.50%
New graduate/intern (first year)	3	7.50%

The results of the demographic questions seen in Table 1 showed that the majority of respondents (62.5%, n = 25) were enrolled or registered nurses, followed by professionals not listed (15%, n = 6) and psychologists (10%, n = 4). Five medical officers partook in this study (12.5% psychiatry registrar/psychiatrist (n = 5)). The majority of respondents worked within a hospital or inpatient department (77.5%, n = 31), whilst some worked in an outpatient department (7.5%, n = 3), day clinic (7.5%, n = 3), or a combination of those listed (7.5%, n = 3). Furthermore, the clinical experience of the respondents varied, with the majority (42.5%, n = 17) having between two and five years of clinical experience, followed by 21+ and 6 to 10 years experience (n = 7, 18%; n = 8, 20%, respectively), and 11 to 15 years experience (10%, n = 4). Only three new graduate (first year) clinicians (7.5%) responded to the questionnaire. Overall, the sample comprises a majority of registered/enrolled nurses, and the sample represents a varying level of clinical experience.

Perceptions of nutrition and primary care

Respondents were then shown a series of statements regarding nutrition and primary care and were then asked whether they agreed or disagreed with the statement(s). Respondents were allowed to choose from the options of Strongly Agree, Agree, Neither Agree nor Disagree, Disagree, and Strongly Disagree in this section of the questionnaire. Table 2 is a collection of this statistical descriptive data. 

**Table 2:** Frequency and Percentage of Responses to Statements (Q4a-f) Total number of participants (N) = 40

a) Statement: "Nutrition has an important part to play in the prevention of mental health illness"	Frequency (n)	Percentage (%)
Strongly Agree	16	40%
Agree	18	45%
Neither Agree nor Disagree	4	10%
Disagree	1	2.5%
Strongly Disagree	1	2.5%
b) Statement: "The primary care team has an essential role in giving dietary advice"		
Agree	22	55%
Strongly Agree	14	35%
Neither Agree nor Disagree	2	5%
Disagree	2	5%
Strongly Disagree	0	0%
c) Statement: "The primary care team has sufficient time to advise patients adequately on nutrition and lifestyle factors impacting their health"		
Strongly Agree	2	5%
Agree	5	12.5%
Neither Agree nor Disagree	12	30%
Disagree	13	32.5%
Strongly Disagree	8	20%
d) Statement: "Advice given will not impact on what people eat"		
Strongly Agree	3	7.5%
Agree	8	20%
Neither Agree nor Disagree	14	35%
Disagree	12	30%
Strongly Disagree	3	7.5%
e) Statement: "Poor nutrition can cause or worsen mental health illness"		
Strongly Agree	21	52.5%
Agree	16	40%
Neither Agree nor Disagree	3	7.5%
Disagree	0	0%
Strongly Disagree	0	0%
f) Statement "I feel confident in my understanding of nutritional psychiatry"		
Strongly Agree	3	7.5%
Agree	15	37.5%
Neither Agree nor Disagree	10	25%
Disagree	11	27.5%
Strongly Disagree	1	2.5%
g) Statement: "I feel that I can adequately advise a patient on their nutritional choices"		
Strongly Agree	6	15%
Agree	18	45%
Neither Agree nor Disagree	11	27.5%
Disagree	4	10%
Strongly Disagree	1	2.5%

The results seen in Table 2 indicate that clinicians either strongly agreed or agreed that nutrition has an important part to play in the prevention of mental illness (85%; n = 34). The data also demonstrated that 90% (n = 36) of respondents believed that the primary care team has an essential role in giving dietary advice, and further, that poor nutrition can cause or worsen mental health illness (Q4e in Table 2) (92.5%; n = 37) as they chose “strongly agree” or “agree” to those respective statements. However, clinicians demonstrated a division on the statement assessing whether the treatment team had sufficient time to advise patients adequately regarding nutrition, as only 17.5% (n = 7) of clinicians either “strongly agreed” or “agreed” with this statement, thereby demonstrating a lack of time as a significant barrier in the delivery of nutritional health information to clients. Furthermore, a question was asked (Q4d in Table 2) to ascertain patient willingness to change their behaviour with nutritional advice (the specific statement being “Advice given will not impact on what people eat”), 27.5% (n = 11) of clinicians agreed or strongly agreed whilst 37.5% (n = 15) disagreed or strongly disagreed, thus illuminating an uncertainty regarding the impact of dietary advice as an intervention in the primary care setting. 

The respondents were also asked whether they believed that poor nutrition could worsen mental illness (Q4e in Table 2), with 92.5% (n = 37) choosing the “agree” or “strongly agree” options (52.5%, n = 21, strongly agreed), with only 7.5% (n = 3) choosing the neutral response, therefore demonstrating a strong consensus. Furthermore, 30% (n = 12) of respondents acknowledged a lack of confidence in their knowledge of nutritional psychiatry (Q4f in Table 2), with only 45% (n = 18) expressing confidence and a further 25% (n = 10) neither agreeing or disagreeing. Similarly, respondents were divided on their ability to adequately advise patients on their nutritional choices (Q4g in Table 2), with 60% (n = 24) demonstrating confidence and a further 27.5% (n = 11) neutral, and 12.5% (n = 5) demonstrating a lack of confidence in providing nutritional advice to patients. 

In the next section of the questionnaire, respondents were given options to identify whether nutritional psychology or nutrition was included in their pre-clinical or post-graduate qualifications, inclusive of professional development, and the amount of time spent studying nutrition. These results are summarised below.

Respondent nutrition education

Respondents, as seen in Table 3, had to select a length of time that nutrition education was included in their pre-clinical or postgraduate education. Respondents were allowed to choose multiple options through checkboxes (Appendix 1) and were also allowed to skip options if they were not relevant to their practice (for example, if they chose not to study at a postgraduate level). The results have demonstrated that a significant number of clinicians (43%; n = 19) have reported having no education or training in nutrition in their pre-clinical studies. Furthermore, the results also showed that the majority of respondents who did report pre-clinical education on nutrition received less than one week (27%; n = 12). 18% (n = 8) of respondents reported one semester (four to six months of study) of nutrition in their preclinical studies. Furthermore, a significant portion of clinicians (29%; n = 11) reported no nutritional training or education in their post-graduate studies, of those that reported such training or qualifications at the post-graduate level. 

**Table 3:** Respondent Identification of Nutritional Education in Pre-clinical and Post-graduate Studies Respondents had the option to report multiple instances of education and/or training. This was particularly relevant for individuals who completed multiple clinical degrees or engaged in various professional development courses throughout their careers. Therefore, the frequencies reflect the number of instances reported, not the number of respondents. The total number of respondents for post-graduate/clinical work is less than the pre-clinical education due to 12 respondents indicating no post-graduate professional qualifications or development.

Length of Study	Pre-clinical Education (Undergraduate/Medical Studies)		Post-graduate/Clinical Work (Such as Professional Development)	
	Frequency (n)	Percentage (%)	Frequency (n)	Percentage (%)
No education or training on nutrition	19	43%	11	29%
Less than one week	12	27%	11	29%
One to three weeks	1	2%	3	8%
One month	2	5%	4	11%
One semester (4-6 months)	8	18%	3	8%
One year	0	0%	2	5%
More than one year	2	5%	4	11%
TOTAL	44	100%	38*	100%

Respondents were then provided with a Likert scale with a rating of 0 (very low importance) to 10 (very high importance, see Appendix 1) in rating their opinions on various issues in regards to nutritional psychiatry. These have been summarised in table 4 as descriptive statistics. 

Respondents were asked to declare their importance of nutritional care in the context of mental health disorders (Q6, Table 4), and a majority of respondents (66%; n = 26) rated the importance at an 8 or above (“very high importance”). More notably, all respondents (100%; n = 40) rated nutritional care at a 5 or above in importance in the context of mental health disorders. However, when asked how important nutritional mental health care is in the context of their workplace (Q7, Table 4), the data shows a more even distribution with 29% (n=11) of clinicians choosing a rating less than 5 (“least important”) in terms of their workplace. The mean result was a 6.7 in level of importance amongst all respondents (n = 40). 

Participants were then asked (Q8, Table 4) to rate their level of nutritional psychiatry from 1 (“very low”) to 10 (“very high”), with a notable observation being that no respondents rated themselves a 9 or 10, with the majority of respondents (25%; n = 10) choosing the moderate score of 6. Furthermore, 54% (n = 21) of clinicians rated their nutritional psychiatry knowledge at a 5 or below (“very low”), with only 23% (n = 9) of respondents choosing a rating of 7 or above. 

**Table 4:** Importance and Knowledge of Nutrition in Mental Health Settings as Reported by Respondents to Questionnaire (Q6-8) Total number of participants (N) = 40

Q6) Importance of Nutritional Care in Mental Health		
Rating	Frequency (n)	Percentage (%)
10 (very high importance)	8	20%
9	6	15%
8	12	31%
7	6	15%
6	5	13%
5	3	8%
4	0	0%
3	0	0%
2	0	0%
1 (very low importance)	0	0%
Q7) Importance of Discussing Nutrition with Patients		
10 (very high importance)	8	20%
9	5	13%
8	6	15%
7	7	18%
6	3	8%
5	1	3%
4	3	8%
3	2	5%
2	3	8%
1 (very low importance)	2	5%
Q8: Current Knowledge of Nutritional Psychiatry		
10 (very high)	0	0%
9	0	0%
8	2	5%
7	7	18%
6	10	25%
5	4	13%
4	5	10%
3	5	13%
2	3	8%
1 (very low)	4	10%

Clinical application of nutritional psychiatry

Table 5 shows clinician responses to prescription or nutritional advice provided in mental health settings. A large proportion of respondents (45%; n = 18) refer their patients to a dietitian, with only 13% (n = 5) regularly prescribing nutritional supplements as part of their mental health care. Further, approximately 23% (n = 9) do not prescribe supplements nor advise their patients to seek a nutritionist. 

**Table 5:** Responses on the Prescription or Nutritional Supplement Advice towards Patients in Mental Health Care Total number of participants (N) = 40

Q9: Clinical Prescription/Advice of Nutritional Supplements for Mental Health		
	Frequency (n)	Percentage (%)
Yes, regularly	5	13%
Yes, rarely	6	15%
Unsure	2	5%
No, but I refer the patient to a dietician	18	45%
No	9	23%

Participants were also asked to choose any dietary recommendations which they may have prescribed or advised to their patients as seen in Table 6. Of those that responded to this optional question (n = 24), the “Mediterranean Diet” (29%; n = 7) was the most popular, together with “other” diets not listed. Similarly, the “low-carb” diet is also a popular nutritional suggestion, with 25% (n = 6) choosing this option. No clinician chose the “ketogenic diet” from the list.

**Table 6:** Dietary Recommendations as Reported by Respondents Note: This question was optional, and responses were provided by 24 of the 40 total participants.

Q10: Dietary Recommendations in Clinical Mental Health Practice		
Selection	Frequency (n)	Percentage (%)
Mediterranean diet	7	29%
Vegetarian diet	1	4%
Vegan diet	3	13%
Ketogenic diet	0	0%
Low-carb diet	6	25%
Other	7	29%
Total	24	100

The questionnaire then asked the clinicians to identify through a list, or identify their own mental health conditions where they have specifically included a nutritional discussion. Note that some conditions such as obesity were included as these may be present within mental health consultations and settings, and respondents were permitted to identify multiple conditions. The results, seen in Table 7, showed that obesity was the most frequently associated condition (18%; n=25) where a nutritional discussion took place, closely followed by depression and eating disorders at 14% (n = 20) each. Psychosis was the least selected (2%) from the list within the questionnaire. 

**Table 7:** Clinician Nutritional Discussion of Various Mental Health Conditions ADHD: Attention Deficit Hyperactivity Disorder; ADHS: Attention Deficit Hyperactivity Syndrome Respondents could select more than one condition; hence why the total exceeds the total number of respondents. Therefore, the frequencies reflect the number of instances reported, not the number of respondents.

Q11. Conditions Addressed with Nutritional Discussions		
Condition	Frequency (n)	Percentage (%)
Symptoms of anxiety	15	11%
Symptoms of depression	20	14%
Psychosis	3	2%
Eating disorders	20	14%
Personality disorders	5	4%
ADHD/ADHS	8	6%
Obsessive compulsive disorder	5	4%
Obesity	25	18%
Metabolic comorbidities	20	14%
Prevention of comorbidities	18	13%
Other-bipolar disorder	1	1%
Other-autism spectrum disorder	1	1%
Other-mood disorder	1	1%
Total	142	100%

When asked about perceived barriers to nutritional management in mental health consumers (table 8), the most common barriers reported by clinicians included a lack of services (such as dietitians) at 22% (n=27), with time constraints and patient willingness to engage in nutrition following closely at 21% (n=26). Similarly, lack of knowledge of nutrition and patient financial constraints received 17% (n=21) each, with the remaining barriers being less common.

**Table 8:** Barriers to Mental Health Nutritional Management Note: Respondents could select more than one barrier, hence why the total exceeds the total number of respondents; Therefore, the frequencies reflect the number of instances reported, not the number of respondents.

Q12: Barriers to Mental Health Nutritional Management		
Factor	Frequency	Percentage
Time constraints	26	21%
Lack of knowledge and/or understanding of nutrition	21	17%
Lack of services eg. allied health support (dietitians)	27	22%
Patient willingness to engage with nutritional discussion	26	21%
Financial constraints of patients	21	17%
Cultural	1	1%
Lack of encouragement of treatment options based on nutrition	1	1%
Other - Focus on medication management	1	1%
TOTAL	124	100%

A question was then asked to ascertain the frequency of diet conversations with their patients (Table 9), with the data demonstrating that 66% (n = 25) surveyed clinicians included nutritional conversations almost all the time with rating of 7 or above, with 20% (n = 8) of respondents choosing a rating of 10 (“always”). Only 5% (n = 2) stated that they “never” discuss diet with their patients (a rating of 1). 

**Table 9:** Frequency of Health Professional Nutrition Discussions with Patients

Q13: Health Professional Nutrition Discussions with Patients		
Rating	Frequency (n)	Percentage (%)
10 (always)	8	20%
9	4	13%
8	6	15%
7	7	18%
6	3	8%
5	2	3%
4	3	8%
3	2	5%
2	3	8%
1 (never)	2	5%
Total	40	100

Furthermore, participants were asked whether they would consider a referral to a nutritionist if a nutritional deficiency was discovered (Table 10), with the majority of respondents (90%; n = 35) indicating that they would, with only a small portion (10%; n = 4) who chose “maybe” as their selection for this particular question.

**Table 10:** Frequency of Respondents who would Consider a Referral to a Nutritionist

Q14: If you identify a patient with a nutritional deficiency, would you consider a referral to a dietitian and/or nutritionist?		
Response	Frequency (n)	Percentage (%)
Yes	35	90%
Maybe	4	10%
No	0	0%
Total	39	100%

Moreover, respondents were then asked about the values of utilising nutritional psychiatry within their approach (Table 11), and the most common value (24%; n = 33) was to “encourage healthier lifestyle choices”, closely followed by “reducing the risk or managing metabolic syndrome" (23%; n = 32), with “preventing chronic disease" at 20% (n = 28).

**Table 11:** Values of Including Nutritional Psychiatry in Clinical Settings Respondents could select more than one factor; hence why the total exceeds the total number of respondents. Therefore, the frequencies reflect the number of instances reported, not the number of respondents.

Q15: What do you think is the value of including nutritional psychiatry in your approach?		
Factor	Frequency (n)	Percentage (%)
To prevent weight gain	26	19%
To reduce the risk or manage metabolic syndrome	32	23%
Prevent medication mismanagement / side effects	18	13%
Encourage healthier lifestyle choices	33	24%
Prevention of chronic diseases (eg. cardiovascular)	28	20%
Other - "holistic care"	1	1%
Other - "mood/anxiety"	1	1%
TOTAL	139	100%

Finally, respondents were then asked to identify whether they would attend a nutritional psychiatry training session if one was offered (Table 12), with an overwhelming majority (92.5%; n = 37) stating “yes”. Conversely, only three respondents (7.5%) stated “no”. 

**Table 12:** Respondent Willingness on Attending Nutritional Psychiatry Training

Q16: If there was a training module available for nutritional psychiatry, would you consider enrolling?		
Response	Frequency (n)	Percentage (%)
Yes	37	92.5%
No	3	7.5%
Total	40	100

## Discussion

This study included a total of 40 mental health clinicians, with the majority comprising nursing staff (62.5%, n = 25) were registered or enrolled nurses), with most respondents employed in an inpatient hospital setting (77.5%, n = 31). More importantly, this study highlighted that 85% (n = 34) of surveyed clinicians agreed that nutrition plays a crucial role in the prevention and/or treatment of mental illness, with the importance of nutrition and mental health is similarly highlighted in the growing body of literature [15-17]. This is particularly relevant in mental health settings as research in the gut-brain axis has demonstrated the association between the gastrointestinal tract, microbiome, and the central nervous system, which together can influence the mental state of the individual [18]. However, despite the acknowledgement of the importance of nutrition in mental health, 64% of the combined barriers identified by respondents in this study demonstrated that they lacked sufficient time (21%), lack of nutrition services (eg: nutritionists) (22%), and patient willingness to engage in nutrition management (21%). Overcoming these barriers would be pivotal in supporting the growth of nutritional psychiatry, particularly through policy and strategic changes such as advocating nutritional discussion in psychiatric consultations and providing incentives to support patients in accessing nutritional psychiatric interventions. 

Furthermore, this study examined the self-rated knowledge and practices of mental health clinicians regarding nutritional psychiatry, and the findings revealed that the respondent clinicians had a low to moderate level of knowledge and understanding of nutritional psychiatry. This is highlighted with the majority of respondents (25%; n = 10) choosing the moderate score of 6, and 54% (n = 21) of clinicians rated their nutritional psychiatry knowledge at a 5 or below (“very low”). Interestingly, one of the factors which may indicate a reason for this lack of knowledge in nutritional psychiatry among mental health clinicians is the lack of nutritional psychiatric and nutrition education and training in their pre-clinical and graduate studies. This study found that 43% (n = 19) of the respondents had no education or training in nutrition in their undergraduate studies, and 29% (n = 11) had no nutrition education in their postgraduate studies. Only 5% (n = 2) of the respondents reported a year or more of education in their pre-clinical education, indicating that those two respondents may have chosen to specialise in nutrition in their undergraduate studies. Morkl et. al's review of the literature identified the lack of clinician education in nutrition as a concern, which is further evidenced by this study [19]. Similarly, another study by Morkl et. al found that the majority of their psychiatric clinicians lacked nutritional literacy, which is further evidenced by this study [20]. These findings therefore suggest that nutrition education does not seem to be a priority or fundamental curriculum requirement, despite further evidence demonstrating the benefits of nutritional psychiatry in preventing and treating mental health disorders [14,15]. Moreover, respondents demonstrated a keen interest in improving their nutritional psychiatry knowledge, as 92.5% (n = 37) of the clinicians reported that they would attend a nutritional psychiatry training session if this was provided, thereby highlighting the recognition of the importance in nutrition on psychiatry by the respondents. 

Additionally, this study also revealed the frequency and conditions where nutrition was discussed by the respondent clinicians; the data showed that the clinicians varied in their nutritional discussions with patients, with 66% (n = 25) rating at of 7 or above, demonstrating there may be different preferences and/or barriers (such as communication and time, as previously discussed) which may explain this data. Furthermore, the survey data also showed that of the different conditions in which the clinicians reported a nutritional discussion, the most common was obesity (18%; n = 25), followed by depression and eating disorders (14%; n = 20 each), and the prevention of metabolic comorbidities (14%; n = 20). However, some mental health conditions which have demonstrated a nutritional benefit such as anxiety disorders and Attention Deficit Hyperactivity Disorder (ADHD) were not as highly reported (11%; n = 15; 6%; n = 8, respectively), potentially demonstrating a lack of awareness on the clinical implications of nutrition management of these conditions [21]. 

Furthermore, the study results shown in this research highlight the gaps and barriers in mental health nutritional psychiatry, as well as show the benefits and role of nutrition in mental health clinical settings. The questionnaire was able to capture a high level of respondent interest in gathering further training and professional development in nutritional psychiatry, if offered by their workplace and/or tertiary institution, thereby adding to the discourse and literature on nutritional psychiatry. However, as the questionnaire was self-administered, the sample size reflected was limited by the willingness of the participants to complete the questionnaire and partake in this study and, therefore, may not reflect the diversity of the mental health clinical community in New South Wales, Australia. The reliability of this research could be enhanced if more mental health clinicians were willing to participate in this research and potentially shorten the questionnaire to facilitate this. For example, future questionnaires in this area could focus on the key themes of practical application of nutritional psychiatry in clinical settings and barriers to implementation, as these were areas which elucidated some interesting insights. In addition, further questionnaires could also gather information from the patient perspective and potential longitudinal studies may examine patient outcomes following nutritional interventions in psychiatric care. 

Recommendations

Based on the findings of our study, there are several recommendations that could be made in supporting the use and integration of nutritional psychiatry in mental health clinical settings.

Incorporate Nutritional Education into Clinical Training

As our study has revealed the identified gaps in nutritional education in clinical training, it is recommended that undergraduate and postgraduate medical, nursing, and allied health programs include curricula based on nutrition and its relationship with mental health. This could support new and existing clinicians in upgrading their knowledge and equipping them in integrating nutritional advice in the psychiatric care. 

Development of Professional Development Programmes

Our study showed an overwhelming level of support in continuing education and training in this area, and therefore it is recommended that professional development programs focused on nutritional psychiatry are offered to existing staff which would support them in integrating such practices in their care. 

Increase Access to Nutritional Services

The lack of nutritional services was highlighted as an important barrier by our respondents, therefore, we recommend that mental health facilities and healthcare systems be offered the services of dieticians and nutritionists who could support and facilitate the mental health teams. 

## Conclusions

This study found that mental health clinicians recognise the importance of nutrition in mental health settings in maximising patient outcomes. However, it also revealed that several challenges exist within mental health settings for nutritional care such as lack of time, confidence, knowledge, nutritional services, and patient willingness to include nutritional interventions within their mental health plan. Interestingly, this study revealed the lack of nutritional education within pre-clinical and post-graduate programmes, and, more importantly, an overwhelming interest in the mental health clinical community to partake in education on nutritional psychiatry. This, therefore, is an important implication for policy and practice, such as providing sufficient time and integrating nutritional psychiatry throughout mental health clinical practice as well as providing affordable and reliable clinical education in nutritional psychiatry. This study demonstrates an important addition to the discourse in nutritional psychiatry, particularly in analysing clinical education in nutritional psychiatry in New South Wales, Australia, and assessing the perceptions of clinicians on nutritional mental health care, whilst also highlighting potential areas for further research in nutritional interventions and/or pre-clinical and graduate education programs in clinical nutritional psychiatry.

## Appendices

 Appendix 1: Questionnaire

**Table 13:** Appendix 1: Questionnaire

1	What is your clinical role?
	Mark only ONE box.
	General Practitioner (GP)
	Psychiatry Registrar
	Psychiatrist
	Registered / Enrolled Nurse
	Psychologist
	Occupational Therapist (OT)
	Other allied health professional not listed
2	What kind of institution do you currently (or have last) work/ed at?
	Check all that apply.
	Hospital / Inpatient Department
	Mental Health outpatient department
	Day Clinic
	Rehabilitation Center
	Private Practice
	Educational Setting
3	How many years of clinical experience do you have?
	Mark only one box.
	New graduate / intern (First year)
	2 to 5 years
	6 to 10 years
	11 to 15 years
	16 to 20 years
	21+ years
	I have no clinical experience

4	Please select if you Agree or Disagree with the following statements:
	Check all that apply.

Statement	Strongly Agree	Agree	Neither Agree or Disagree	Disagree	Strongly Disagree
Nutrition has an important part to play in the prevention of mental health illness					
The primary care team has an essential role in giving dietary advice					
The primary care team has sufficient time to advise patients adequately on nutrition and lifestyle factors impacting their health					
Advice given will not impact on what people eat					
Poor nutrition can cause or worsen mental health illness					
I feel confident in my understanding of nutritional psychiatry					
I feel that I can adequately advise a patient on their nutritional choices					

5. Please identify any FORMAL (including professional development courses) education you have received on nutrition/nutritional psychiatry as part of your pre-clinical education (undergraduate/medical studies) and/or post-graduate/clinical work (such as professional development). Check all that apply.

Statement	Pre-clinical education (Undergraduate / medical studies)	Post-graduate / Clinical Work (such as professional development)
No education or training on nutrition		
Less than one week		
One to three weeks		
One month		
One semester (4-6 months)		
One year		
More than one year		

6. From 1 to 10, in your opinion, how important is nutritional care in the context of mental health disorders?
Mark only one box.

	1	2	3	4	5	6	7	8	9	10	
Very Low Importance											Very High Importance

7. From 1 to 10, in your workplace or institution, how important is discussing nutrition with your patients?
Mark only one box

	1	2	3	4	5	6	7	8	9	10	
Very Low Importance											Very High Importance

8. From 1 to 10, how would you rate your current knowledge of nutritional psychiatry?
Mark only one box.

	1	2	3	4	5	6	7	8	9	10	
Very Low											Very High

9.	Have you ever prescribed or advised a patient to take nutritional supplements or specific dietary foods in improving their mental health condition(s)? Mark only one box.
	Yes, regularly
	Yes, rarely
	Unsure
	No, but I refer the patient to a dietician
	No
10	Please click on any of the following diets which you may have prescribed or advised patients to follow as part of your clinical practice or therapeutic interactions with patients in mental health settings. You may add your own if it is not listed.
	Check all that apply.
	Mediterranean diet
	Vegetarian diet
	Vegan diet
	Ketogenic diet
	Low-carb diet
	Other:
11	For which conditions mainly, have you included nutritional discussion in your management approach?
	Check all that apply.
	Symptoms of anxiety
	Symptoms of depression
	Psychosis
	Eating disorders
	Personality disorders
	ADHD / ADHS
	Obsessive Compulsive Disorder
	Obesity
	Metabolic comorbidities
	Prevention of comorbidities
	Other:
12	What barriers do you identify in the nutritional management of your mental health consumers?
	Check all that apply.
	Time constraints
	Lack of knowledge and/or understanding of nutrition
	Lack of services eg. allied health support (dietitians)
	Patient willingness to engage with nutritional discussion
	Financial constraints of patients
	Other:

13	From 1 to 10, how often do you usually talk to your patients about their diet?
	Mark only one box.

	1	2	3	4	5	6	7	8	9	10	
Never											Always

14	If you identify a patient with a nutritional deficiency - would you consider a referral to a dietitian and/or nutritionist?
	Mark only one box.
	Yes
	No
	Maybe
15	What do you think is the value of including nutritional psychiatry in your approach?
	Check all that apply.
	To prevent weight gain
	To reduce the risk or manage metabolic syndrome
	Prevent medication mismanagement / side effects
	Encourage healthier lifestyle choices
	Prevention of chronic diseases (eg. cardiovascular)
	Other:
16	IF there was a training module available for nutritional psychiatry, would you consider enrolling?
	Mark only one box.
	Yes
	No

ADHS
